# Sinc-Windowing and Multiple Correlation Coefficients Improve SSVEP Recognition Based on Canonical Correlation Analysis

**DOI:** 10.1155/2018/4278782

**Published:** 2018-04-12

**Authors:** Valeria Mondini, Anna Lisa Mangia, Luca Talevi, Angelo Cappello

**Affiliations:** Department of Electrical, Electronic and Information Engineering (DEI), University of Bologna, Cesena, Italy

## Abstract

Canonical Correlation Analysis (CCA) is an increasingly used approach in the field of Steady-State Visually Evoked Potential (SSVEP) recognition. The efficacy of the method has been widely proven, and several variations have been proposed. However, most CCA variations tend to complicate the method, usually requiring additional user training or increasing computational load. Taking simple procedures and low computational costs may be, however, a relevant aspect, especially in view of low-cost and high-portability devices. In addition, it would be desirable that the proposed variations are as general and modular as possible to facilitate the translation of results to different algorithms and setups. In this work, we evaluated the impact of two simple, modular variations of the classical CCA method. The variations involved (i) the number of canonical correlations used for classification and (ii) the inclusion of a prefiltering step by means of sinc-windowing. We tested ten volunteers in a 4-class SSVEP setup. Both variations significantly improved classification accuracy when they were used separately or in conjunction and led to accuracy increments up to 7-8% on average and peak of 25–30%. Additionally, variations had no (variation (i)) or minimal (variation (ii)) impact on the number of algorithm steps required for each classification. Given the modular nature of the proposed variations and their positive impact on classification accuracy, they might be easily included in the design of CCA-based algorithms that are even different from ours.

## 1. Introduction

A Brain-Computer Interface (BCI) is a system enabling direct communication between the brain and the outside, as it directly translates the recorded neural activity into a control signal for an external device (e.g., a computer, a machine, or a speller) [1]. Among noninvasive systems, electroencephalography- (EEG-) based BCIs are the most widespread [2], and they can rely on four possible electrophysiological sources: slow cortical potentials (SCPs), event-related desynchronization/synchronization (ERD/ERS), event-related potentials (as P300), or Steady-State Visually Evoked Potentials (SSVEPs) [3]. Among these, SSVEP-based BCIs are appealing for their high accuracies and information transfer rate (ITR), thanks to the high signal-to-noise ratio of SSVEPs even without user training [4]. For this reason, SSVEP-based BCIs have been raising increasing attention over the years [5, 6].

SSVEPs are periodic brain signals elicited over the occipital cortex by visual stimulations with frequencies higher than 6 Hz [7]. In case different flickering objects (LEDs, symbols, and squares) are simultaneously presented, an analysis of the SSVEP spectral content permits to reconstruct which stimulus the user is focusing on.

Traditionally used methods perform SSVEP recognition based on power spectral density analysis (PSDA) [7]. In PSDA-based approaches, spectral powers are estimated from the EEG spectrum at the target stimulation frequencies and used as a feature for classification [8–10]. However, PSDA-based methods can suffer from noise sensitivity if few channels are acquired, besides requiring relatively long signal portions (e.g., >3 s) to estimate the spectrum with a sufficient frequency resolution [11–13]. A promising and increasingly used approach, which has recently attracted the interest of researchers [14–17], is the one based on Canonical Correlation Analysis (CCA) [7].

CCA is a multivariate statistical method able to reveal the underlying correlation between two sets of data [18]. For SSVEP recognition, CCA is performed several times between the considered EEG segment and a set of sine-cosine reference signals modeling the pure SSVEP responses to each stimulation frequency [7]. The frequency response showing highest correlation with the analyzed EEG portion is finally recognized as the observed one.

The efficacy of the CCA approach has been widely proven, and its superiority to PSDA in terms of speed, accuracy, and computational load has been shown [19, 20]. For this reason, several CCA variations have been proposed over the years [11–13, 15, 21–26].

Some CCA variations, as [11–13, 15, 21, 23], modified the SSVEP reference signals by including subject-specific features from each user's EEG. The work in [24] enriched the algorithm with incorporating intersubject information from the signals of multiple subjects. In [25], an effort was made towards compensating the natural decrease in signal-to-noise ratio of SSVEPs at higher stimulation frequencies by correcting classification gains based on the shape of individual background EEG. Finally, in [22, 26], CCA was repeated multiple times for each stimulation frequency, each time processing the signal with a different IIR band-pass filter, to combine different aspects of the same EEG response.

Although each introduced variation produced significant increments of classification accuracy, all of them tended to increase the complexity of the algorithm. They indeed either required additional user training, to incorporate information from individual EEG data [11–13, 15, 21, 23], or increased computational load by multiplying the number of CCAs to assess each stimulation frequency [22, 26]. However, we believe that even taking simple procedures and low computational costs may be relevant, especially to favor the spread of low-cost and high-portability devices. In addition, it would be desirable that variations are as general or scalable as possible to facilitate the translation of results to different setups.

Given these premises, this work presents two simple and modular variations based on the classical CCA method. The variations regard (i) the number of correlations considered for classification and (ii) the preprocessing of the signals. We show that both modifications can significantly improve classification accuracy but still leaving the whole procedure training-free and with no (variation (i)) or minimal (variation (ii)) impact on the number of steps required for each SSVEP identification.

## 2. Materials and Methods

### 2.1. The Standard CCA Method for SSVEP Recognition

Canonical Correlation Analysis (CCA) is a multivariate statistical method [18] used to reveal the underlying correlation between two sets of data. Given two sets of random variables** X **∈*ℝ*^*I*_1_×*J*^ and** Y **∈*ℝ*^*I*_2_×*J*^, CCA finds the two corresponding sets** U **=** AX **∈*ℝ*^*I*_1_×*J*^ and** V** =** BY **∈*ℝ*^*I*_2_×*J*^ (linear combination of the original ones through** A **∈*ℝ*^*I*_1_^ and** B **∈*ℝ*^*I*_2_^), called* canonical variables*, so that the correlation between each pair or rows (*U*_*i*_, *V*_*i*_) is maximized:

(1) ρi=max⁡covUi,VivarUivarVi=maxA,B⁡covAXi,BYivarAXivarBYi

with leaving (*U*_*i*_, *V*_*j*_), (*U*_*i*_, *U*_*j*_), and (*V*_*i*_, *V*_*j*_) uncorrelated if *i* ≠ *j*. Each CCA leads to a number of solutions *ρ*_*i*_ equal to the minimum between the numbers of rows in** A **(*I*_1_) and** B **(*I*_2_). The solutions *ρ*_*i*_, sorted in descending order, are called* canonical correlations* and are a measure of the similarity between the two sets of original data.

The use of CCA in the field of SSVEP recognition was first proposed by Lin et al. in [7]. Given *K* stimulation frequencies to be distinguished, CCA is performed *K* times, one for each stimulation frequency *f*_*k*_, between the multichannel EEG signal in** X **∈*ℝ*^*N*_ch_×*J*^ (*N*_ch_ acquired channels, *J* time samples) and a set of sine-cosine reference signals in **Y**_*k*_∈*ℝ*^2*N*_harm_×*J*^ modeling the pure SSVEP responses. Each set **Y**_*k*_ is composed as follows:

(2) Yk=cos⁡2πfktsin⁡2πfktcos⁡2π2fktsin⁡2π2fkt⋮cos⁡2πNharmfktsin⁡2πNharmfkt, t=1Fs,2Fs,…,JFs,

where *f*_*k*_ is the stimulation frequency, *F*_*s*_ is the sampling rate, and *N*_harm_ is the number of harmonics included in the analysis.

Every CCA generates a vector of canonical correlations (*ρ*_*k*1_, *ρ*_*k*2_,…, *ρ*_*k*min(*N*_ch_, 2*N*_harm_)_), of which only the first and largest one, *ρ*_*k*1_, is used as a feature for classification. The analyzed EEG segment in** X** is indeed assigned to the stimulation frequency leading to the maximum correlation *ρ*_*k*1_:

(3) $$\begin{array}{c} f_{target}=\underset{f_{k}}{\max}\;\rho_{k1}. \end{array}$$

### 2.2. Variation 1: Number of Considered Canonical Correlations

Although the efficacy of the CCA method for SSVEP recognition has been widely proven [14, 16] and many variations have been proposed [11–13, 15, 21–27], the majority of approaches consider only the first canonical correlation as a feature for classification. Nevertheless, as already noted by Lin et al. [7], since real EEG signals may be contaminated by noise and show phase transitions, the information might be spread over more than one correlation coefficient.

As a first variation of the algorithm, we evaluated the impact of taking a combination of more than one correlation coefficient as a feature for classification, following preliminary results in [28]. Since the canonical variables in** U** and** V** are estimated so that each couple (*U*_*i*_, *U*_*j*_) and (*V*_*i*_, *V*_*j*_) are uncorrelated for *i* ≠ *j* and the sine-cosine waves in the reference signals **Y**_*k*_ are orthogonal between each other, the information contained in each set of canonical variables will always be in quadrature with respect to the others. For this reason, we propose combining the *N*_corr_ considered correlations with using the Euclidean norm:

(4) $$\begin{array}{c} r_{k}=\sqrt{\overset{N_{corr}}{\underset{i=1}{\sum }}\rho_{ki}^{2}}. \end{array}$$

The resulting combination *r*_*k*_ would be used as a feature for classification in place of the largest canonical correlation *ρ*_*k*1_ only. The number *N*_corr_ can range from 1 to the minimum between *N*_ch_ and 2*N*_harm_, with *N*_ch_ number of acquired channels and *N*_harm_ number of considered harmonics. In this work, we employed *N*_ch_ = 8 EEG channels (see Section 2.4 for details) and *N*_harm_ = 3 harmonics, so we explored the impact of taking all the possible numbers of considered correlations between 1 and 2*N*_harm_.

### 2.3. Variation 2: Preprocessing with Sinc-Windowing

Another possible variation with respect to literature may consist in adding a preprocessing step to the EEG segments before performing CCA. If we exclude the works in [22, 26], employing IIR filter banks, CCA is indeed typically applied without any prefiltering of the EEG signals. Nevertheless, we believe that a narrow-band prefiltering step around the *K* employed stimulation frequencies and their *N*_harm_ harmonics might be useful to increase the signal-to-noise ratio, expectantly enhancing classification accuracy.

As a second variation, we evaluated the influence of such type of prefiltering with using a sinc-windowing implementation. The technique of sinc-windowing consists in the convolution of the analyzed signal with an adequately modulated sinc function. As it is known, the inverse Fourier transform of an ideal rectangular band-pass filter centered in *f*_0_ and with *M* bandwidth is

(5) rectf−f0M+rectf+f0M→F−12Msinc⁡Mtcos⁡2πf0t,

where* f* is the frequency and *F*^−1^ is the inverse Fourier transform. Thus, the filtering around the *f*_*k*_ stimulation frequencies and *N*_harm_ harmonics can be accomplished by means of a convolution with the following function:

(6) $$\begin{array}{c} h\left(\;t\;\right)=2M\mathrm{sinc}\left(\;Mt\;\right)\left(\;\overset{K}{\underset{k=1}{\sum }}\overset{N_{harm}}{\underset{n=1}{\sum }}\cos\left(\;2\pi nf_{k}t\;\right)\;\right), \end{array}$$

where *M* is the bandwidth (in this work, *M* = 1 Hz), *N*_harm_ is the number of harmonics, and *f*_*k*_ are the *K* stimulation frequencies.

### 2.4. Data Acquisition

The EEG was recorded from 8 electrodes (PO7, PO8, PO3, PO4, O1, O2, POz, and Oz), positioned according to the international 10-20 system. The signals were acquired using a Brainbox EEG-1166 amplifier, with a 256 Hz sampling frequency and a 50 Hz Notch filter on.

SSVEP stimulation was provided through four blue LEDs, arranged around a PC monitor. Each LED flickered at a different stimulation frequency (*f*_1_ = 8 Hz, *f*_2_ = 9 Hz, *f*_3_ = 10 Hz, and *f*_4_ = 11 Hz). The four stimulation frequencies were selected before the beginning of the study and were the same for all subjects. All stimulations were provided with a 50 percent duty-cycle. The behavior of the LEDs was controlled by a LabVIEW-Arduino interface.

### 2.5. Experimental Paradigm

Ten healthy volunteers (aged 22 to 26, 4 males and 6 females) participated in the study. All of them had normal, or corrected to, normal vision. During the experiment, the participants sat on a comfortable chair, with their arms relaxed and their head still, approximately 60 cm distant from the PC monitor.

The experiment was organized into runs and the runs were organized into trials. Each participant underwent a total of 4 runs, each comprising 16 trials. Each trial consisted of three subsequent phases: a 1 s* preamble*, a 12 s* stimulation*, and a 2 s* break* period. During the* preamble*, a yellow square appeared on the screen indicating the target LED; then all LEDs started simultaneously flickering during* stimulation*, and the trial ended with a* break* period, where the LEDs shut off and the square disappeared. The order of the target LEDs was randomized and counterbalanced in each run, so that each LED was gazed for the same amount of time. To summarize, each experiment included a total of 4 runs × 16 trials × 12 seconds = 768 seconds of stimulation, that is, 192 seconds for each class.

### 2.6. Performance Evaluation

For each subject, we evaluated the average classification accuracy at the end of each run. To highlight the impact of the two proposed variations (composition of the feature vector and sinc-windowing), all accuracies were recomputed using all the possible combinations of methods, that is, a number of considered correlations from one to *N*_corr_ = 6, with or without sinc-windowing. To evaluate the influence of considering different lengths of EEG signal for SSVEP recognition, all accuracies were recomputed with considering signal portions ranging from 0.5 s to 5 s, although the detailed results of statistical tests will be reported only in the case of a 1.5 s window length.

Another commonly used measure of BCI performance, encompassing the concepts of speed, accuracy, and number of choices, is the measure of information transfer rate (ITR), expressed in bit/min. For reasons of completeness, ITR was also provided, and it was computed according to [29]

(7) ITR bit/min=60Tlog2⁡N+p log2⁡p+1−plog2⁡1−pN−1,

where *N* = 4 is the number of choices, *p* is the classification accuracy (expressed between 0 and 1), and *T* is the epoch duration (in seconds).

For the sake of comparison with other CCA-based literature methods that might be related to ours, we finally recomputed classification accuracies with the method of Chen et al. in [26], employing IIR filter banks, while we omit the comparison with [22] as not reasonably adaptable to our setup.

### 2.7. Statistical Analyses

At first, we compared each accuracy to chance level. The value of chance level was obtained by running the simulations as descripted in [30] in the case of a 4-class BCI and taking the upper bound of the confidence interval at *α* = 1% significance, as an analytical expression of chance level was not available for the multiclass case. As concerns statistical comparison between methods, we had to account for the fact that multiple data came from the same subject; that is, the samples could not be assumed to be completely independent. For this reason, instead of using paired-samples *t*-test to compare each method against the others, we ran all evaluations as post hoc tests of a repeated-measures ANOVA. The ANOVA design included both the factors “method” (the within-subject factor) and “subject,” thus taking into consideration all dependencies among data. Post hoc tests were carried out using Bonferroni correction. The use of parametric statistical tests was justified by the normality of data distributions, as confirmed by the application of a preliminary Kolmogorov-Smirnov test.

## 3. Results

**Figure 1:**
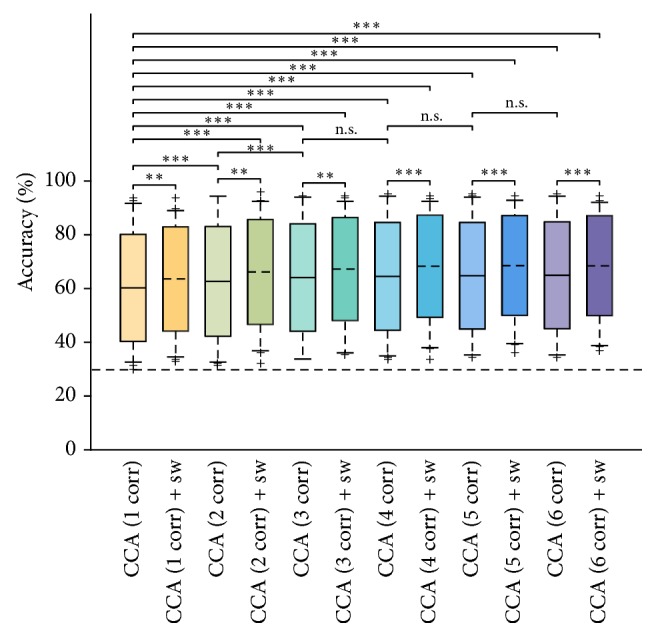
A boxplot showing the classification accuracy distributions for all the considered combinations of methods. The asterisks mark statistical significance, ^*∗∗*^*p* < 0.01 and ^*∗∗∗*^*p* < 0.001, while “n.s.” indicates the absence of significance. The horizontal, dashed line marks the upper confidence interval for chance level (*α* = 1%).

**Figure 2:**
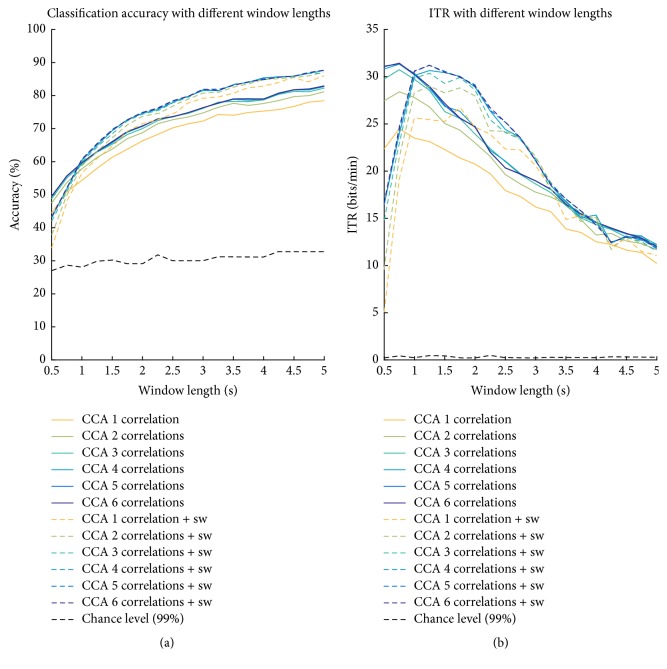
Grand average across subjects and runs of the classification accuracies (a) and ITR (b) for all the considered methods. The black-dashed line indicates the upper confidence interval of the chance level (*α* = 1%) (a) and its corresponding ITR (b). Note that chance level is slightly different for the different time windows, as the consideration of a larger time window implies a reduction in the number of trials per class.

The classification accuracies of each subject, run, and method are detailed in Table 1 and summarized in Figure 1. The last two rows of Table 1 indicate the average and peak increment of each method with respect to standard CCA (first column). All the obtained accuracies were significantly higher than chance, as the upper bound of the confidence interval for chance level (with a significance of *α* = 1%) in this particular setup was 30.27%. In Table 2, the results of the post hoc comparisons (Bonferroni-corrected) between each pair of methods are reported. In Figure 2, the accuracy curves of all the considered methods, evaluated with different windows lengths, are shown. In order to avoid redundancies, the detailed ITRs for each subject, run, and method are omitted, as they can be easily computed from the accuracy results in Table 1 and according to (7). Nevertheless, Table 3 reports the average and peak ITR of each combination of methods, together with the average and peak increment in ITR with respect to classical CCA, in the same manner as reported in the last rows of Table 1.

Both proposed variations were able to significantly improve classification accuracy. As regards variation 1, the results in Tables 1 and 2 and Figure 1 clearly show how the consideration of more than one canonical correlation significantly increases classification accuracy in both the sinc-windowing and no-sinc-windowing conditions. Nevertheless, while accuracy significantly increases (*p* < 0.001, both with or without sinc-windowing) when switching from one to two canonical correlations or from two to three canonical correlations (*p* < 0.001, in the no-sinc-windowing condition), the increment generally becomes insignificant when taking four, five, or six canonical correlations, with respect, for example, to three. As concerns variation 2, that is, the inclusion of a prefiltering step around the *K* stimulation frequencies and *N*_harm_ harmonics by means of sinc-windowing, the results show how this kind of preprocessing always outperformed (with statistical significances ranging from *p* < 0.001 to *p* < 0.01) the corresponding version without processing. Accordingly, when variation 1 and variation 2 were combined, classification accuracy was a fortiori significantly (*p* < 0.01 or *p* < 0.001) increased with respect to the standard CCA method. To give an example, the accuracies obtained with using four canonical correlations and sinc-windowing were averagely increased by 8.20% with respect to the standard CCA method, with a peak increment of even 31.25% (in S08, run 2).

When varying the length of the EEG portions used to recognize the SSVEPs, the behavior of the proposed variations on classification accuracy tended to be confirmed, with the only exception of the 0.5 s window length (Figure 2). While the consideration of more than one canonical correlation always outperformed the use of the largest one only, the positive impact of sinc-windowing emerged only for window lengths greater than 0.5–1 s.

When finally recomputing accuracies with the filter bank CCA method proposed in [26], we confirm that the latter performed significantly (*p* < 0.001) better than standard CCA. However, the increase in accuracy produced by [26] was not statistically different from some of our proposed variations. Notably, accuracy results obtained with the combinations of four, five, or six canonical correlations and sinc-windowing processing were not statistically different from the results of filter bank CCA [26].

## 4. Discussion

Our results show how the simple consideration of more than one canonical correlation can significantly improve the achievable accuracy without any increment of computational load. As already suggested by Lin et al. [7], real EEG signals are affected by noise and can show phase transitions; therefore the information might be spread over more than one correlation coefficient.

From a theoretical point of view, if the EEG signals (in the** X** matrix) were almost unaffected by noise and shared the same phase across electrodes (i.e., the rows in** X**), then the consideration of only the first canonical correlation would be sufficient to capture the majority of information. As indeed the sine-cosine waves in the rows of each **Y**_*k*_ are an orthogonal basis, CCA would be able to find that particular linear transformation of **Y**_*k*_ able to explain the behavior of the SSVEP response in** X** through maximizing the correlation between a linear combination of** X** (the EEG signals) and **Y**_*k*_, without leaving information behind. However, as** X** is a multichannel set of data, if we suppose that the SSVEP response might show a different phase across electrodes (i.e.,** X** rows), then at least a second set of canonical variables would be needed to explain the data, and the second set (*U*_2_, *V*_2_) would contain a complementary information with respect to (*U*_1_, *V*_1_). If we further suppose that, at the same EEG location, the different harmonics of the same SSVEP response might show different delays between each other, then at least another set of canonical variables (*U*_3_, *V*_3_) would be needed to capture the information of the SSVEP response not included in the first two sets.

We suggest that all the above-introduced suppositions are likely to be true in real EEG signals. Supposing indeed that the SSVEP response is generated in a limited area of the occipital cortex, it will undergo different delays to reach the different locations of electrodes, due to a delay in spatial transmission. However, we suggest that the second supposition also is reasonable in real EEG. Given indeed the origin of SSVEP in the occipital cortex, the signal has to pass through multiple tissue layers (fluids, bone, and skin) before reaching each EEG location. This is likely to produce phase distortion between different frequency components, besides the well-known spatial blurring effect.

The above-described interpretation fits the experimental results well; indeed the accuracy significantly increased when switching from one to three canonical correlations. We consequently suggest that the consideration of more than one canonical correlation permits to encompass a more complete information on the investigated frequency *f*_*k*_, and this finally translates in an increased accuracy, revealed in almost every subject and run. From the third set of canonical variables on, we hypothesize that the amount of information described by each correlation depends on each user's individual characteristics, for example, the amount of delay across different harmonics and electrodes, as well as the differential amplitude of the SSVEP response between different harmonics of the same stimulation frequency. According to this hypothesis, from the fourth canonical correlation on, there would not be a group effect anymore, and this would explain why the accuracy increments in the experimental data are not significant anymore.

Besides recommending the consideration of more than one canonical correlation, our results also highlight the positive impact of prefiltering before CCA performance. The presence of a filtering stage around the *K* stimulation frequencies and related *N*_harm_ harmonics may have permitted to enhance the SSVEP response from the background EEG, and this finally translated in a significantly increased accuracy in every considered comparison between corresponding versions of the method, with or without prefiltering. The idea of exploiting band-pass filters to enhance different SSVEP components had been already introduced in the works of Chen et al. [26] and Islam et al. [22], suggesting the use of IIR filter banks. However, both algorithm implementations in [22, 26] were proposed to perform multiple prefilterings of the same EEG portion, thus multiplying the number of CCAs to assess each stimulation frequency. Despite being able to produce a significant increase in classification accuracy, this implies a multiplication of the total number of steps required in each SSVEP recognition, with a related sensible increment of computational load. Besides being a novelty with respect to literature, the implementation of the prefiltering by means of sinc-windowing has the advantage of being able to filter multiple frequency components in one single step, by simply modulating the composition of the convolved function. This implies that one more single step is added to each SSVEP recognition independently from the number *K* of stimulation frequencies or *N*_harm_ considered harmonics, thus overall remaining computationally light.

A potential limitation of the sinc-windowing technique might be related to the length of the considered signal portions, due to the Gibbs truncation effect [31]. As indeed shown in Figure 2, while for segment lengths longer than 1 s sinc-windowing increased the achievable accuracy, it turned to have even a negative impact when considering a short signal portion of 0.5 s. Figure 2(b) integrates the information of Figure 2(a), reminding that an increase in window length may cause a decrease in ITR (as deducible from (7)), in case the accuracy increase is not enough to contrast the decrease of number of classifications per time. It results that the maximum ITR can be achieved, for each considered comparison, with window lengths of 1.25–1.5 s, while the positive impact of sinc-windowing is most evident up to 2.5–3 s window length. As final comment on the sinc-windowing technique, it might be noted that its efficacy was generally confirmed despite the closeness of the chosen stimulation frequencies (8, 9, 10, and 11 Hz).

As regards the obtained accuracies in absolute terms, our results are in line with literature regarding multiclass SSVEP recognition with the standard CCA technique [7, 14, 20, 26, 32], although a subject-specific calibration of the stimulation frequencies and/or their duty cycles [33] could have further increased the performances. In addition, we verified that the combination of our proposed variations could produce the same accuracy increments as other CCA-related methods in literature and particularly the same improvements as filter bank CCA of Chen et al. [26].

As a final comment, we believe that, beyond making a comparison of our methods to literature, the main aim and contribution of this work were giving a systematic study of the effect of two simple, modular, and computationally light variations of the standard CCA algorithm. These proposed variations might be intended as modular “algorithm bricks” and might be flexibly translated to the design of CCA-based algorithm that is even different from ours in order to increase the overall accuracy.

## 5. Conclusion

In this work, we evaluated the impact of two simple and modular variations of the CCA algorithm in a 4-class SSVEP recognition setup. The two variations involved (i) the number of considered canonical correlations and (ii) the inclusion of a narrow-band prefiltering step around the employed stimulation frequencies and related harmonics by means of sinc-windowing technique. Our results indicate that even simple consideration of more than one canonical correlation can significantly improve accuracy, without any increment of computational load. Notably, there were significant increases in accuracy when switching from one to three canonical correlations, while the increments were not significant from the fourth canonical correlation on. An additional narrow-band prefiltering permitted to gain up to 7-8% of accuracy on average, with peaks of 25–30%, with respect to classical CCA. A further advantage of sinc-windowing implementation is that it permits the enhancement of multiple frequency components in one single step, by simply modulating the composition of the sinc-function. Given the modular nature of the proposed variations and the significant increments in accuracy, regardless of whether the variations were used separately or, even more, in combination, together with the minimal computational costs, we believe that they could easily represent valid integrations to be included in future CCA-based designs.

## Figures and Tables

**Table 1:** Detailed accuracies (%) for each subject and run and for all combinations of methods, with a window length of 1.5 s. The last rows of the table summarize the average and peak accuracy of each combination, together with the average and peak increment with respect to classical CCA.

	CCA (1 corr)	CCA (2 corr)	CCA (3 corr)	CCA (4 corr)	CCA (5 corr)	CCA (6 corr)	CCA (1 corr) + sw	CCA (2 corr) + sw	CCA (3 corr) + sw	CCA (4 corr) + sw	CCA (5 corr) + sw	CCA (6 corr) + sw
S01												
* Run 1*	92.2	96.1	96.1	96.9	96.9	96.9	91.4	93.8	95.3	95.3	96.1	96.1
* Run 2*	92.2	93.8	95.3	95.3	95.3	96.1	89.8	93.8	92.2	93.0	93.0	93.0
* Run 3*	95.3	96.1	96.1	96.9	96.1	96.1	95.3	97.7	96.1	96.1	96.1	94.5
* Run 4*	94.5	96.1	95.3	95.3	95.3	95.3	89.8	94.5	93.0	91.4	92.2	92.2

S02												
* Run 1*	85.2	87.5	90.6	90.6	90.6	90.6	85.2	91.4	90.6	90.6	91.4	91.4
* Run 2*	78.9	79.7	79.7	78.9	78.9	78.9	80.5	85.2	87.5	85.2	84.4	84.4
* Run 3*	82.8	85.2	85.2	87.5	86.7	86.7	87.5	90.6	92.2	93.0	92.2	92.2
* Run 4*	88.3	89.8	89.8	90.6	90.6	90.6	87.5	90.6	88.3	91.4	92.2	93.0

S03												
* Run 1*	80.5	87.5	86.7	88.3	88.3	88.3	80.5	78.1	79.7	78.9	78.9	77.3
* Run 2*	78.9	82.0	82.0	82.0	82.0	82.0	80.5	77.3	75.8	75.8	75.0	75.0
* Run 3*	74.2	79.7	82.0	82.8	82.8	82.8	71.9	74.2	78.1	78.9	78.9	78.1
* Run 4*	82.8	85.9	86.7	87.5	87.5	87.5	83.6	88.3	87.5	85.2	85.2	85.9

S04												
* Run 1*	75. 8	79.7	82.0	82.8	82.0	82.8	85.9	85.9	85.2	85.9	86.7	85.9
* Run 2*	64.8	68.8	70.3	70.3	71.9	71.9	79.7	80.5	81.3	82.0	82.8	83.6
* Run 3*	69.5	73.4	73.4	72.7	74.2	75.8	78.9	83.6	84.4	86.7	84.4	84.4
* Run 4*	68.0	66.4	71.1	70.3	70.3	71.1	83.6	83.6	84.4	85.2	82.8	82.0

S05												
* Run 1*	64.1	68.0	72.7	74.2	74.2	74.2	73.4	79.7	82.0	82.8	84.4	83.6
* Run 2*	76.6	78.9	79.7	79.7	79.7	79.7	74.2	81.3	82.8	87.5	86.7	87.5
* Run 3*	61.7	66.4	66.4	67.2	68.0	68.0	63.3	66.4	71.9	72.7	75.0	75.8
* Run 4*	69.5	75.8	78.1	78.9	78.9	78.9	81.3	77.3	80.5	82.8	82.8	82.0

S06												
* Run 1*	60.9	66.4	66.4	67.2	67.2	68.0	64.1	63.3	64.8	69.5	71.1	72.7
* Run 2*	64.1	60.9	61.7	61.7	63.3	63.3	63.3	64.1	70.3	71.1	68.8	68.8
* Run 3*	54.7	59.4	60.9	61.7	60.9	60.9	63.3	63.3	63.3	64.1	64.1	64.1
* Run 4*	59.4	58.6	61.7	62.5	64.1	65.6	55.5	60.9	64.1	68.0	69.5	71.1

S07												
* Run 1*	52.3	57.8	57.8	57.8	57.0	57.0	51.6	56.3	53.1	54.7	51.6	50.8
* Run 2*	39.8	42.2	44.5	44.5	43.00	43.8	45.3	51.6	50.8	54.7	57.0	56.3
* Run 3*	38.3	41.4	40.6	40.6	40.6	40.6	40.6	36.7	41.4	42.2	43.0	43.8
* Run 4*	43.00	43.8	43.8	45.3	45.3	45.3	48.4	53.9	53.9	57.8	59.4	58.6

S08												
* Run 1*	46.9	50.0	53.1	53.9	53.9	53.9	52.3	57.8	61.7	60.9	61.7	60.2
* Run 2*	40.6	44.5	47.7	50.8	51.6	51.6	59.4	64.1	67.2	71.9	68.8	68.8
* Run 3*	46.1	49.2	53.9	53.1	53.9	53.9	50.0	55.5	59.4	60.9	63.3	63.3
* Run 4*	42.2	43.0	47.7	48.4	48.4	48.4	46.1	53.1	58.6	60.2	57.0	58.6

S09												
* Run 1*	39.1	39.8	42.9	41.4	43.7	43.8	34.4	39.1	39.1	40.6	40.6	39.8
* Run 2*	35.9	32.0	34.4	35.9	37.5	37.5	40.6	38.3	35.9	39.1	39.8	39.1
* Run 3*	39.1	38.3	38.3	37.5	35.9	35.9	35.9	38.3	36.7	38.3	40.6	39.8
* Run 4*	34.4	33.6	34.4	34.4	37.5	36.7	33.6	32.8	36.7	34.4	36.7	37.5

S10												
* Run 1*	30.5	32.8	34.4	35.2	35.2	35.2	39.1	39.8	39.8	39.1	41.4	41.4
* Run 2*	42.2	46.9	47.7	47.7	47.7	47.7	45.3	50.8	48.4	46.9	47.7	48.4
* Run 3*	32.0	35.2	35.9	35.9	35.9	35.9	35.9	40.6	41.4	42.2	43.0	43.0
* Run 4*	35.9	39.8	43.0	43.8	44.5	44.5	39.9	40.6	43.8	44.5	46.9	46.1

*Average*	*61.3*	*63.8*	*65.3*	*65.7*	*65.9*	*66.1*	*64.7*	*67.4*	*68.5*	*69.5*	*69.8*	*69.8*
* Peak*	*95.3*	*96.1*	*96.1*	*96.9*	*96.9*	*96.9*	*95.3*	*97.7*	*96.1*	*96.1*	*96.1*	*96.1*

*Average Δ*	—	*2.48*	*3.92*	*4.37*	*4.60*	*4.76*	*3.38*	*6.03*	*7.14*	*8.20*	*8.49*	*8.4*
* Peak Δ*	—	*7.03*	*8.59*	*10.2*	*10.9*	*10.9*	*18.8*	*23.4*	*26.6*	*31.3*	*28.1*	*28.1*

**Table 2:** *p* values from the post hoc comparisons between each pair of methods. The asterisks mark statistical significance: ^*∗*^*p* < 0.05, ^*∗∗*^*p* < 0.01, and ^*∗∗∗*^*p* < 0.001.

	CCA (1 corr)	CCA (2 corr)	CCA (3 corr)	CCA (4 corr)	CCA (5 corr)	CCA (6 corr)
CCA (1 corr)	*—*	*p* < 10^−5^^*∗∗∗*^	*p* < 10^−9^^*∗∗∗*^	*p* < 10^−9^^*∗∗∗*^	*p* < 10^−10^^*∗∗∗*^	*p* < 10^−10^^*∗∗∗*^
CCA (2 corr)	*—*	*—*	*p* < 10^−4^^*∗∗∗*^	*p* < 10^−5^^*∗∗∗*^	*p* < 10^−5^^*∗∗∗*^	*p* < 10^−5^^*∗∗∗*^
CCA (3 corr)	*—*	*—*	*—*	*p* = 0.32	*p* = 0.13	*p* = 0.017^*∗*^
CCA (4 corr)	*—*	*—*	*—*	*—*	*p* = 1	*p* = 0.017^*∗*^
CCA (5 corr)	*—*	*—*	*—*	*—*	*—*	*p* = 0.90
CCA (6 corr)	*—*	*—*	*—*	*—*	*—*	*—*

	CCA (1 corr) + sw	CCA (2 corr) + sw	CCA (3 corr) + sw	CCA (4 corr) + sw	CCA (5 corr) + sw	CCA (6 corr) + sw

CCA (1 corr) + sw	*—*	*p* < 10^−3^^*∗∗∗*^	*p* < 10^−5^^*∗∗∗*^	*p* < 10^−6^^*∗∗∗*^	*p* < 10^−6^^*∗∗∗*^	*p* < 10^−6^^*∗∗∗*^
CCA (2 corr) + sw	*—*	*—*	*p* = 0.21	*p* < 10^−3^^*∗∗∗*^	*p* < 10^−3^^*∗∗∗*^	*p* = 0.0022^*∗∗*^
CCA (3 corr) + sw	*—*	*—*	*—*	*p* = 0.041^*∗*^	*p* = 0.053	*p* = 0.19
CCA (4 corr) + sw	*—*	*—*	*—*	*—*	*p* = 1	*p* = 1
CCA (5 corr) + sw	*—*	*—*	*—*	*—*	*—*	*p* = 1
CCA (6 corr) + sw	*—*	*—*	*—*	*—*	*—*	*—*

	CCA (1 corr)	CCA (2 corr)	CCA (3 corr)	CCA (4 corr)	CCA (5 corr)	CCA (6 corr)

CCA (1 corr) + sw	*p* = 0.0014^*∗∗*^	*p* = 1	*p* = 1	*p* = 1	*p* = 1	*p* = 1
CCA (2 corr) + sw	*p* < 10^−8^^*∗∗∗*^	*p* = 0.0015^*∗∗*^	*p* = 0.22	*p* = 0.77	*p* = 1	*p* = 1
CCA (3 corr) + sw	*p* < 10^−10^^*∗∗∗*^	*p* < 10^−4^^*∗∗∗*^	*p* = 0.0025^*∗∗*^	*p* = 0.0082^*∗∗*^	*p* = 0.018^*∗*^	*p* = 0.042^*∗*^
CCA (4 corr) + sw	*p* < 10^−10^^*∗∗∗*^	*p* < 10^−4^^*∗∗∗*^	*p* < 10^−4^^*∗∗∗*^	*p* < 10^−3^^*∗∗∗*^	*p* < 10^−3^^*∗∗∗*^	*p* < 10^−3^^*∗∗∗*^
CCA (5 corr) + sw	*p* < 10^−10^^*∗∗∗*^	*p* < 10^−6^^*∗∗∗*^	*p* < 10^−4^^*∗∗∗*^	*p* < 10^−4^^*∗∗∗*^	*p* < 10^−3^^*∗∗∗*^	*p* < 10^−3^^*∗∗∗*^
CCA (6 corr) + sw	*p* < 10^−10^^*∗∗∗*^	*p* < 10^−6^^*∗∗∗*^	*p* < 10^−4^^*∗∗∗*^	*p* < 10^−4^^*∗∗∗*^	*p* < 10^−3^^*∗∗∗*^	*p* < 10^−3^^*∗∗∗*^

**Table 3:** Average and peak ITR (bits/min) of each combination of methods, together with average and peak increment with respect to classical CCA, with a window length of 1.5 s.

	CCA (1 corr)	CCA (2 corr)	CCA (3 corr)	CCA (4 corr)	CCA (5 corr)	CCA (6 corr)	CCA (1 corr) + sw	CCA (2 corr) + sw	CCA (3 corr) + sw	CCA (4 corr) + sw	CCA (5 corr) + sw	CCA (6 corr) + sw
Average ITR	22.28	25.05	26.30	26.86	26.97	27.18	25.24	28.30	29.30	30.42	30.55	30.45
Peak ITR	66.11	68.00	68.00	69.99	69.99	69.99	66.11	72.10	68.00	68.00	68.00	68.00
Average Δ ITR	*—*	2.77	4.01	4.58	4.68	4.89	2.96	6.01	7.02	8.14	8.27	8.17
Peak Δ ITR	*—*	11.21	10.71	12.60	12.60	12.60	20.33	20.33	21.90	24.51	25.55	24.31
